# Supplementary material for: Multiplex PCR Assay for Simultaneous Identification of Five Types of Tuna (Katsuwonus pelamis, Thunnus alalonga, T. albacares, T. obesus and T. thynnus)
Source: Foods. 2022 Jan 20;11(3):280. doi: 10.3390/foods11030280 (PMC8834044; doi:10.3390/foods11030280)
Supplement: Supplementary file 1 [file foods-11-00280-s001.zip › foods-1552138-supplementary.pdf]

# Supplementary material

(A)

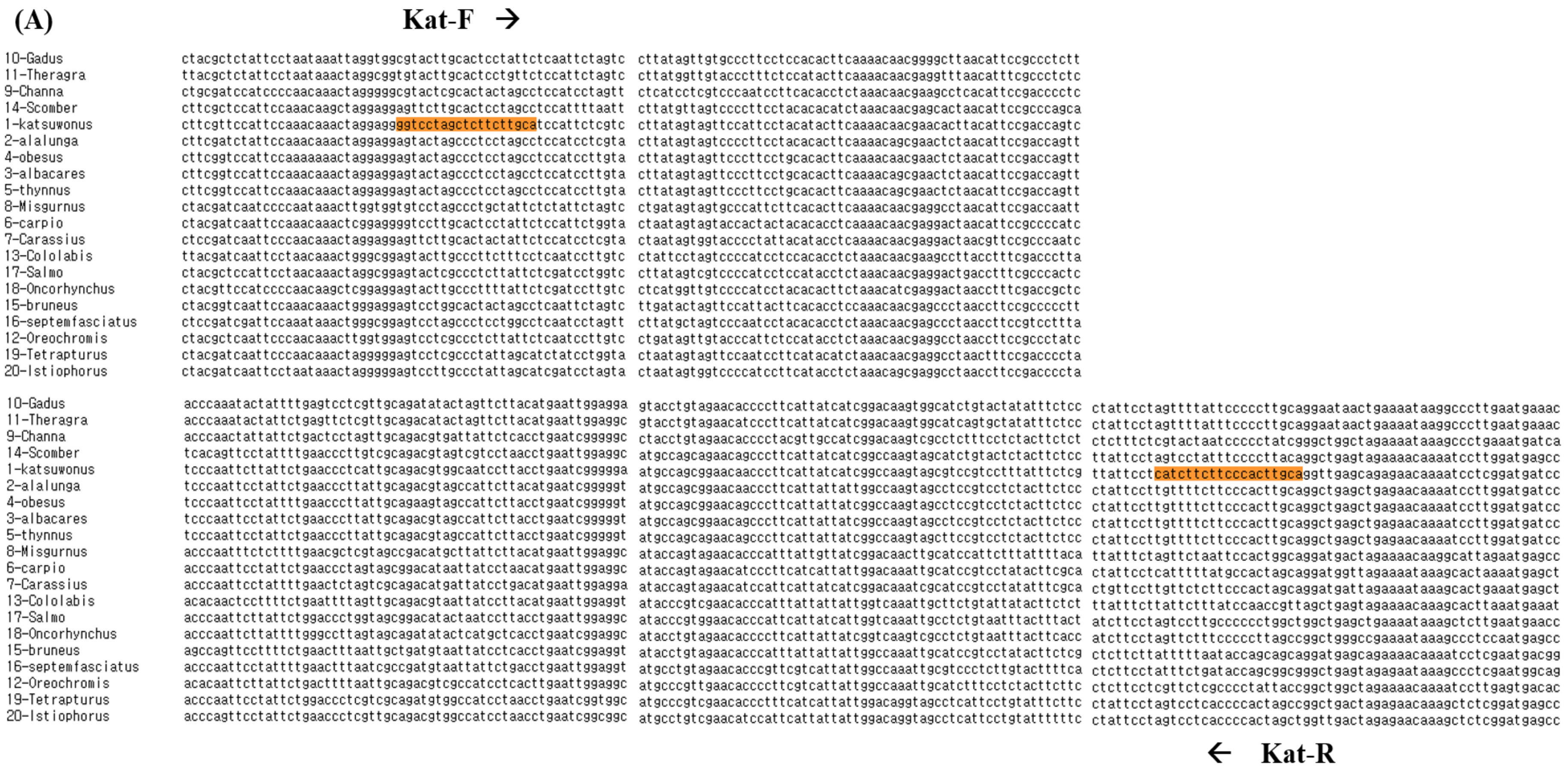

**(B)**

**Ala-F →**

```
10-Gadus             cacccatatttcacctacaaagacctgctcggctttgctgtaatgcttctgggcttaacc gccctcgccctcttcgcacctaatttacttggagatccagataatttcacccctgctaac
11-Theragra          cacccatatttcacgtacaaagacctgcttggctttgctgtaatacttctaggcttaacc gccctcgcccttttcgcacctaatttgcttggagatccagataatttcacgcctgctaac
9-Channa             caccoctacttctcctataaagacctcctgggcttcgctatccttctaatctccctcatc tccctgtccctattcgcccccaacctccttggagacccagacaacttcacccccgctaac
14-Scomber           cacccatacttcacctacaaagacctccttggctttgccgtcctcctcgtggccctctcc tctctcgcactcttctctcccaacctcttgggagaccccgacaatttcacaccagctaac
1-katsuwonus         cacccatacttctcctacaaagacctccttggcttcgcaattctactagtagctctcgct tctctagcactattctcccctaacctcctaggagacccagacaatttcacgccagccaac
2-alalunga           cacccatacttctcttacaaagacctccttggtttcgtgatcctgctagtggcactcgcc tctctagcactattctcccctaacctcctgggagacccagacaacttcacccctgccaac
4-obesus             cacccatacttctcttacaaagacctccttggtttcgtgattctgctagtagcactcgcc tctctagcactattctctcccaacctcctaggagacccagacaacttcacccctgccaac
3-albacares          cacccatacttctcttacaaagacctccttggtttcgtgatcctgctagtagcactcgcc tctctagcactattctcccccaacctcctaggagacccagacaacttcacccctgccaac
5-thynnus            cacccatacttctcttacaaagacctccttggtttcgtgatcctgctagtagcactcgcc tctctagcactattctcccctaacctcctaggagatccagacaacttcacccctgccaac
8-Misgurnus          caccoctacttttcttacaaagacctacttggatttgctgtagttctgctcgccctcaca tccttgtcattgttctcccccaatctcctaggagacccagacaattttacccccgcaaac
6-carpio             cacccgtacttctcatacaaagacctccttgggttcgtaattatactcctagctcttaca ctactagcactattctcccctaacttactaggagacccagaaaacttcacccccgcaaac
7-Carassius          cacccatacttttcatacaaagacctccttgggttcgtgattatactactagccctcaca ctcctggcattattctccccaaacctttt aggagacccagaaaacttcactccagccaat
13-Cololabis         cacccatacttctcttataaagatattttaggcttcgcagccctgtttgtagcactaatc tccttagctttattctcccccaacctcttaggtgacccagataatttcacccctgctaac
17-Salmo             caccottacttctcatataaagacctcctcggatttgtagccatactacttggcctaaca tccttagctctattcgcacccaacctcctcggggacccagacaattttacacctgccaac
18-Oncorhynchus      caccottacttctcatacaaagatctcctaggattcgtagccatactcctaggcctaaca tccttagctctttttgcaccaaatctcctaggggacccagacaattttacgcctgccaac
15-bruneus           cacccatacttttcttacaaagacctactagggtttgcagccttacttattgcacttaca tccctagcactattctcccccaacctattaggagacccagacaatttcacccctgccaac
16-septemfasciatus   cacccatacttctcatacaaagacctcttaggatttgcagccctacttattgcacttaca tcattagccctatttacaccaaacctactaggagacccagacaacttcaccccagccaac
12-Oreochromis       caccoctacttttcttacaaagacttattaggcttcgcaattcttttaattgcccttatt tctttagccctcttctcccccaatctacttggtgatcctgacaacttcacccccgcaaac
19-Tetrapturus       caccoctacttctcctacaaagacctcctaggttttgccgcccttctgatcggcctaacc tcccttgctcttttctcccctaatctgcttggtgatcctgacaactttacgccagcaaac
20-Istiophorus       caccoctacttctcctacaaagacctcttaggttttgccgcccttctaattggcctaacc tccctcgcccttttctcccctaacctgctcggtgaccccgacaactttacgccggcaaac

10-Gadus             cccatcgttaccccacctcatgttaagcctgaatgatatttcttgtttgcctacgccatc ctacgctctattcctaataaattaggtggcgtacttgcactcctattctcaattctagtc
11-Theragra          cccatcgttacccctcctcatgttaagcccgagtgatatttcttgtttgcctacgccatc ttacgctctattcctaataaactaggcggtgtacttgcactcctgttctccattctagtc
9-Channa             ccgctagtcacaccaccccacatcaaaccagagtggtacttcctatttgcctacgccatt ctgcgatccatccccaacaaactagggggcgtactcgcactactagcctccatcctagtt
14-Scomber           ccaatagtaacccctcctcatattaagcctgaatgatacttcctatttgcatacgcaatt cttcgctccattccaaacaagctaggaggagttcttgcactcctagcctccattttaatt
1-katsuwonus         cccatggttacgcctccccacattaaacccgaatggtacttcctatttgcctacgcaatt cttcgttccattccaaacaaactaggaggggtcctagctcttcttgcatccattctcgtc
2-alalunga           ccaatggtcaccccacctcacattaaacctgaatgatacttcctatttgcttacgcaatt cttcgatctattccaaacaaactaggaggagtactagccctcctagcctccatcctcgta
4-obesus             cccatggttaccccacctcacattaaacctgaatgatatttcctatttgcatacgcaatt cttcggtccattccaaaaaaactaggaggagtactagccctcctagcctccatccttgta
3-albacares          cctatggttaccccacctcacattaaacctgaatgatatttcctatttgcatacgcaatt cttcggtccattccaaacaaactaggaggagtactagccctcctagcctccatccttgta
5-thynnus            ccaatggttactccacctcacattaaacctgaatgatatttcctatttgcatacgcaatt cttcggtccattccaaacaaactaggaggagtactagccctcctagcctccatccttgta
8-Misgurnus          ccactagtaacacccccccacatcaagccagagtggtatttcttatttgcctacgcgatt ctacgatcaatccccaataaacttggtggtgtcctagccctgctattctctattctagtc
6-carpio             cctctagttacaccaccccacatcaaaccagaatgatacttcctatttgcctacgccatc ctacgatcaattccaaacaaactcggaggggtccttgcactcctattctccattctggta
7-Carassius          cccctggttactcccctcatattaaaccagagtgatatttcctgtttgcctacgccatt ctccgatcaattcccaacaaactaggaggagttcttgcactactattctccatcctcgta
13-Cololabis         cccttagttactccccccacattaaaccagaatgatacttcctattcgcgtacgccatt ttacgatcaattcctaacaaactgggcggagtacttgcccttctttcctcaatccttgtc
17-Salmo             cccctagttactccacctcatatcaagcctgaatgatacttcctattcgcctacgcaatc ctacgctccattcctaacaaactaggcggagtactcgccctcttattctcgatcctggtc
18-Oncorhynchus      cccctagtgaccccacctcatattaaacccgaatgatacttcctattcgcttacgcaatc ctacgttccatccccaacaagctcggaggagtacttgcccttttattctcgatccttgtc
15-bruneus           cccctcgtcacgcctcctcacattaagcctgaatgatacttcttattcgcttacgccatc ctacggtcaattccaaacaaactgggaggagtcctggcactactagcctcaattctagtc
16-septemfasciatus   ccccttgtcacccctccccacatcaaacccgaatggtacttcttattcgcgtacgccatc ctccgatcgattccaaataaactgggcggagtcctagccctcctggcctcaatcctagtt
12-Oreochromis       cctctagttacccctccccacattaaacccgaatggtacttcttatttgcctacgccatc ctacgctcaattcccaacaaacttggtggagtcctcgccctcttattctcaatccttgtc
19-Tetrapturus       ccccttgtaacccctcctcacattaaacctgagtgatatttcctctttgcctatgccatc ctacgatcaattcccaacaaactagggggagtcctcgccctattagcatctatcctggta
20-Istiophorus       cccctcgtaacccctcctcacattaaacctgaatgatatttcctctttgcctacgccatc ctacgatcaattcctaataaactagggggagtccttgccctattagcatcgatcctagta
```

**← Ala-R**

**(C)**

**Alba-F →**

```
17-Salmo              ggtattctaatccagacgccctgaggatttactggtgcaattatcctaatgattgcacac  ggtctcgcctcctcagcactgttctgtttagccaacacaagctacgaacgcacacacagc
18-Oncorhynchus       ggtattttaattcaaacaccttgaggatttactggtgcaattattctcataatcgcacac  ggccttgcctcctcagcgctattctgcttagccaatactagctacgaacgcacccacagc
8-Misgurnus           ggaatcctaattcaaaccccatgaggctttacaggtgcaatcattttaataattgcccac  ggattagtatcatccgcacttttctgtctggctaataccgcctacgagcgaacacatagc
6-carpio              ggaatcctaattcaaaccccatgaggattctcaggagcaatcatcctaataattgctcac  ggactagtatcctcggcacttttctgcttagccaacacagcctatgaacgaacccatagc
7-Crucian             ggaattttaatccaaaccccatgaggattttcaggagcaattattttaataattgcccac  ggattagtctcctcagcactattttgcttagccaacacagcttatgaacgaacacacagc
10-Gadus              ggaattttaattcagaccccttggggatttacaggggctttgattctaataattgctcat  ggcctggcctcatcagctttattctgtcttgctaataccaattacgaacgaactcacagc
11-Theragra           ggaattttaattcaaaccccttggggatttacaggggctttaattctaataattgctcat  ggcctagcctcatcagctttattctgtcttgctaataccaattacgaacgaactcatagc
13-Cololabis          gcaatccttattcaaacaccatgaagcttcacaggagccctgatccttataattgcccac  ggactaacatcatccgccctattttgcctagccaatactaattatgagcgtacccatagc
9-Channa              ggcattctaattcaaacccccctgaggtttcacaggcgctctagtcctcataattgcccac  ggattaacatcatccgccttattctgcctagccaatactaactacgaacgcacgcacaac
15-bruneus            ggaatcttaatccaaacccccctgaggattcacaggagccttaatccttataatcgcccac  gggttaacttcttctgctctattctgtttagccaatacaaactacgaacgaacacacagc
16-septemfasciatus    ggtatcctaattcaaaccccttgagggtttacaggagccctaattcttataatcgcccac  ggattaacatcctccgccctattctgcttagctaacacaaactacgaacgcacacacagc
12-Oreochromis        ggcattctaatccaaaccccctgaggctttacaggcgccctcattctaataatcgcacac  ggtttaacttcctccgccctcttctgcttagctaacacgaactacgaacgaacacacagc
19-Tetrapturus        ggaattctcattcaaacccccctgaggctttacaggagcccttattcttataatcgcacat  ggtttaacctcctccgccttattctgcttggcaaataccaattatgaacgaacacatagc
20-Istiophorus        ggaattctcattcaaaccccctgaggctttacaggagcccttattcttataattgcacat  ggtctaacctcctccgctttattctgcttagcaaacaccaactatgaacgaacccacagc
14-Scomber            ggaatccttatccaaacccccctgaggttttacaggagccctaatcctaataattgcacat  ggcctaacatcttccgccctcttctgtctagcaaacaccaactatgaacgaacacatagc
1-katsuwonus          ggcattttaatccaaacaccctgaggctttacaggagccctcatcctcataatcgcacac  ggattaacttcttccgcactattctgcctagcgaacactaactacgaacgaacccacagc
2-alalunga            ggtatcctaatccaaacaccctgaggttttacaggggcccttatccttataatcgcccac  ggactaacctcctccgctcttttctgcttagccaatactaactatgaacgaacacatagc
5-thynnus             ggcattctaatccaaacaccctgaggctttacgggagcccttatccttatgattgcccac  ggactaacctcctccgctcttttctgcttagccaacactaactacgaacgaacacatagc
3-albacares           ggcattctaatccaaacaccctgaggctttacgggggccctcatcctcatgattgcccac  ggacttacctcctccgctcttttctgcttagccaatactaactacgagcgaacacatagc
4-obesus              ggtattctaatccaaacaccctgaggctttacgggggcccttaccctcatgattgcccac  ggattaacctcctccgctcttttctgcttagccaacactaactacgagcgaacacatagc

17-Salmo              cgaaccatgctactagctcgaggaatacaaataattgtcccctaataaccacttgatga
18-Oncorhynchus       cgaaccatactacttgcccgaggaatacaaataattctccccttaataaccacttggtga
8-Misgurnus           cgaactataattcttgcccgaggactacaaataatcttcccactaaccgcagtgtgatga
6-carpio              cgaacaataattctcgcccgaggcctacaagtaattttcccactaactgcagtatgatgg
7-Crucian             cgaacaataatccttgcccgaggactacaaattatttttccactaaccgcagtatggtga
10-Gadus              cggacaatacttttagcccgaggacttcaaattgctctcccacttatgaccacatgatga
11-Theragra           cggacaatacttttagcccgaggacttcaaattgctctcccacttatgaccacatgatga
13-Cololabis          cgaacaatggttcttgcacgaggcctacagatagtccttccaattataaccacctgatga
9-Channa              cgaacaataattctagcccgaggcctacaaatagttctacccctggcgaccacctgatgg
15-bruneus            cgaaccatacttctagcgcgcggcctacaaatcgtacttcccctgataacagcctgatga
16-septemfasciatus    cgaactatactcctagcacgtggcctacaaattatcttgcccctaataacagcctgatga
12-Oreochromis        cgaactataattttagcgcgaggactccaaatggttttaccccctaataaccgcatgatgg
19-Tetrapturus        cgaaccatgcttttagcacgaggccttcagatagtcctccctttaatggccacgtgatga
20-Istiophorus        cgaaccatgcttctagcccgaggccttcaaatagtcctcccgctaatagccacatgatga
14-Scomber            cgaacaatagtgttagcacgaggcctgcaaatagtactccccttataactacctgatga
1-katsuwonus          cgaacaatagtcctagcacgaggccttcaaatagtcctacctttaataacaacatgatga
2-alalunga            cgaacaatggttctggcacgaggactgcaaatagtattacccttataacaacatgatga
5-thynnus             cgaacaatggttctggcacgaggactgcaaatagtattacccttataacaacatgatga
3-albacares           cgaacaatggttctggcacgaggactgcagatagtgctgccccttataacaacatgatga
4-obesus              cgaacaatggttctggcacgaggcctgcagatagtactgccccttataacaacatgatga
```

**← Alba-R**

**(D)**

**Obe-F →**

| | | | |
|---|---|---|---|
| 10-Gadus | cttaatttaggtttagctgttcccctctgattagcaaccgtccttatcggtatacgaaat | cagccaactcatgcactaggtcatttccttccagagggcactcctacagccctaattcct | attctaattattatcgaaacaattagtctattcattcgccctctcgctttaggcgttcgg |
| 11-Theragra | cttaatttaggtttagctgttcccctctgattagcaactgtccttatcggtatacgaaat | cagccaactcatgcactaggccacttcctcccagaagggacccccacagccttaattcct | attctaattattatcgaaacaattagtctattcattcgccctctcgctttaggcgttcgg |
| 15-bruneus | cttaacttaggatttgcagtccccctctgattagctactgttatcattggaatacgaaac | caaccaaatcacgcactaggtcaccttctaccagaaggcacccctaatctcctaatccct | atactcattgtcatcgaaacaatcagcctatttatccgccccctggccttaggtgtacga |
| 16-septemfasciatus | cttaacctaggcttcgccgtccccctttgattggcaactgtcattattggaatgcgaaac | caaccaaatcatgctctaggacaccttctaccagaaggaacccctagtctcctaatccca | atactaattattatcgaaacaattagcctattcatccggcccctggccctgggtgtacga |
| 13-Cololabis | ataaatataggtcttgccgtcccactttgattagcaacagtaattattgggatacgaaac | caacccacccacgcactaggtcacctcttacctgaaggaacacctactctgttaatccct | gtactaattattatcgaaacaattagcctttttattcgacctttagctcttggagttcga |
| 12-Oreochromis | ctcaacatagccttcgctgtacccctctgacttgctacagtcattattggtatacgaaac | caacctacacatgcgctaggccaccttctgccagaaggcactcctaccctcttgatccct | gtcctaatcattatcgaaacaattagcctatttattcggcccctcgcacttggagttcga |
| 17-Salmo | ctaaatataggccttgcagtccccttatgactcgcaacagtaattattggcatgcgaaac | caacccaccgccgccctaggccatctcctgccggaaggaactcccgtcccactaatcccg | gttcttattattatcgagacaattagcctttttatccgccccctcgcccttggtgtacga |
| 18-Oncorhynchus | ctaaatatgggcctcgcagtcccactgtggcttgctacagtaattatcggcatacgaaac | cagcctacggccgccctcggccatttattgcctgaaggaaccccgttccactgatccca | gtactgatcattatcgaaacaattagcctttttatccgccccctcgcccttggcgtacga |
| 14-Scomber | ctaaatatgggcttagccgtccccttttgactagcaacagttatcattgggatgcgaaac | caaccaactgagtcactaggccacctcctgccagaaggaacgcccaccctcctcattcca | gtcctaatcattatcgaaactattagcctattcattcgtccccttgcccttggagttcga |
| 1-katsuwonus | ctaaacatgggccttgcattccctctatgactagcaacagttatcatcggcatgcgaaac | caaccaacagaagccttaggccacctccttcccgaaggaactcctacacttcttattccc | gtcctaattattatcgaaacaattagcctatttatccgaccattagcactaggagtacgg |
| 2-alalunga | ctcaatatgggacttgcattccctctttgattagcaactgttattattggcatgcgaaac | cagccaacagaagccctgggccaccttcttccagaagggacacctacgctacttattcca | gtactaattgtcatcgaaacaattagcttattcatccgacccttagcacttggagtgcgg |
| 4-obesus | ctcaacatggg**acttgcattccccctatgg**ttagcaactgttattattggcatgcgaaac | caaccaacagaagccctaggccacctccttccagaaggaacacctacactacttatccca | gtactaattgtcatcgaaacaattagcttattcatccgacccctagcacttggagtccgg |
| 3-albacares | ctcaacatgggacttgcattcccctctgattagcaactgttattattggtatgcgaaac | caaccaacagaagccctaggccatctccttccagaaggaacacctacactacttatccca | gtactaattgtcatcgaaacaattagcttattcatccgacccttagcacttggagtgcgg |
| 5-thynnus | ctcaacatgggacttgcattcccctctgattagcaactgttattattggtatgcgaaac | caaccaacagaagccctaggccaactccttccagaaggaacacctacactactcatccca | gtactaattgtcatcgaaacaattagcttattcatccgacccttagcacttggagtgcgg |
| 8-Misgurnus | ctaaatataggatttgcagttccactatgactcgccacagttattattggtatacgtaat | caaccaacagttgcactaggtcaccttctcccagaaggaacccctatccccctgattccc | gtattaattattatcgaaacaattagcctatttattcgccccctagccctgggtgtccga |
| 6-carpio | ctcaatatgggatttgccgtacctctatgactcgctactgtaattattgggatacgaaat | caaccaacagttgccctaggacacctactaccagaaggaacacccattccactgatccca | gtactaattatcatcgaaacaattagcctatttatccgcccattagccctgggagtccga |
| 7-Crucian | ctcaatatgggatttgctgtaccactatgacttgctacagtaattatcggaatgcgaaat | caaccaacagtagctttaggtcacctgttaccagaaggtacacccatcccactgattcca | gtactaattattatcgaaacaattagtctatttattcgaccattagccctaggagtccga |
| 9-Channa | ataaacatggggctcgccgtccccctttgattggcgactgtaattatcggcctccgaacc | caaccaacacatgcactaggacatctcctccccgaagggacccctacacttctaatcccc | gtcctcatcattatcgaaaccattagcctcttcattcgccccttggccctgggggtccga |
| 19-Tetrapturus | ttaaacatgggccttgcagttcccctctgacttgcaacagttattattggaatacgaaat | caaccaactcatgccctaggtcacctcctccctgaaggaaccccaacccttctaatccct | gttctcattattatcgaaactatcagcctattcattcgccctcttgccctaggggtccga |
| 20-Istiophorus | cttaacatgggccttgcagttcccctctgacttgcaacagtcattattggtatgcgaaac | caaccaacccacgccctaggtcacctcctccctgaaggaaccccaacccttctaatccct | gttcttattattatcgaaacaatcagcctattcatccgccctcttgccctaggagtccga |

| | | |
|---|---|---|
| 10-Gadus | cttacagctaatctcacagcaggtcatttactaattcatctaatttcctcagcagtcttt | gttcttatacctataatacctgcagtcgctattcttacagcagttctccttctattactt |
| 11-Theragra | cttacagctaatctcacagcaggtcatttactgattcatctaatttcctcagcagtgttt | gttctcataccaataatacctgcagttgctattcttacagcagttctccttctactactc |
| 15-bruneus | ctaactgctaacctaacagcaggccacctgctcattcaattaatctccacagctgtattt | gtactcctaccactcataccaaccgtagctattcttacagcaacagtcctagttctttta |
| 16-septemfasciatus | ctaaccgctaacctaacagctggtcacctgcttattcaactaatctcaacagctgccctt | gtgcttctaccacttttacccacagtagctatcctcacagcaactgtcctagttctttta |
| 13-Cololabis | cttacagctaacctaacagcaggccaccttttaattcagctaattgctactgccgccttc | gttcttgcctcaacagcacccactctagctatccttactactatactacttttctcctc |
| 12-Oreochromis | ctaaccgcaaatcttacagctggtcatctcttaattcaactcatcgccaccgccgccttc | gttcttcttccccttatacctacagtagcgatcctaactgcagtattactctttctgcta |
| 17-Salmo | ctcacagccaatctcacagcaggccacctcctaattcaactaatcgctacagcagccttt | gtcctcatacctatcatacctacagtagcaatcctaacttctatcgtcctcttcctactt |
| 18-Oncorhynchus | cttacagccaatctcacagcaggccaccttcttatccaactaattgctacagcagccttt | gttcttctacctataatacctacggtagcaatccttacttctattgtcctctttctactc |
| 14-Scomber | ctaacagccaaccttacagccggccaccttctaattcaactaatcgcaacagctgcatct | gttcttctcccactaatgcccactgttgccattctaaccgggattttattattccttta |
| 1-katsuwonus | ctaacagccaatctcacagccggccatcttctaattcagctaatcgccacagccgcaact | gttctccttccactaatgccaaccgtagcaatcctaacagcaaccatcctcttccttcta |
| 2-alalunga | ttaacagccaacctaacggctggacatcttctaattcaactaatcgctacagcagcaact | gtccttctaccactaatgccaactgtagctatcctaacagcaaccctacttttccttcta |
| 4-obesus | tttacagctaacctaacggctggacatcttttaattcaactaatcgctacagcagcaact | gtccttctaccactaatgccaa**ctgtggcaatcctaacagc**aaccctacttttccttcta |
| 3-albacares | ttaacagctaacctgacggctggacatcttttaattcaactaatcgctacagcagcaact | gtccttctaccactaatgccaaccgtagcaatcctaacagcaaccctgcttttccttcta |
| 5-thynnus | ttaacagccaacctaacggctggacatcttttaattcaactaatcgctacagcagcaact | gtccttctaccactaatgccaactgtggcaatcctaacagcaaccctacttttccttcta |
| 8-Misgurnus | ctcacggctaacctaaccgctggacatctattaattcaactaatcgccaccgccgtattt | gttcttctcccaataatgcccacagtagctattttaaccgccaccgttttatttctatta |
| 6-carpio | ctaacagccaatctaaccgcaggccacctgttaatccaactcatcgccacagctgtattt | gttctcctaccaataataccaacagtagcaatcctaaccgctgccgtactcttcctcctc |
| 7-Crucian | ctcacagccaacttaactgcaggccaccttctaattcaacttatcgccacagctgtattt | gttctcctaccaataataccaacagtagcaattctaaccgccacagtattatttttacta |
| 9-Channa | ctgacagccaatctcactgctggtcacctcctaatccaactaattgccactgctgccttc | gtcctcctcccattaatgcccacagtagccatcctaacggccacccttttattcctactc |
| 19-Tetrapturus | cttactgccaacttaacagctggccacctgctaattcaacttattgcaacagccgccttc | gtactccttccccttatgccaaccgtagctattttaacagccacactcctattcctactc |
| 20-Istiophorus | ttgacagctaacttaacagctggccacctacttattcaacttattgcaacagccgccttt | gtactcctccccccttataccaaccgtagctattttaacagccacactcctattcctactt |

**← Obe-R**

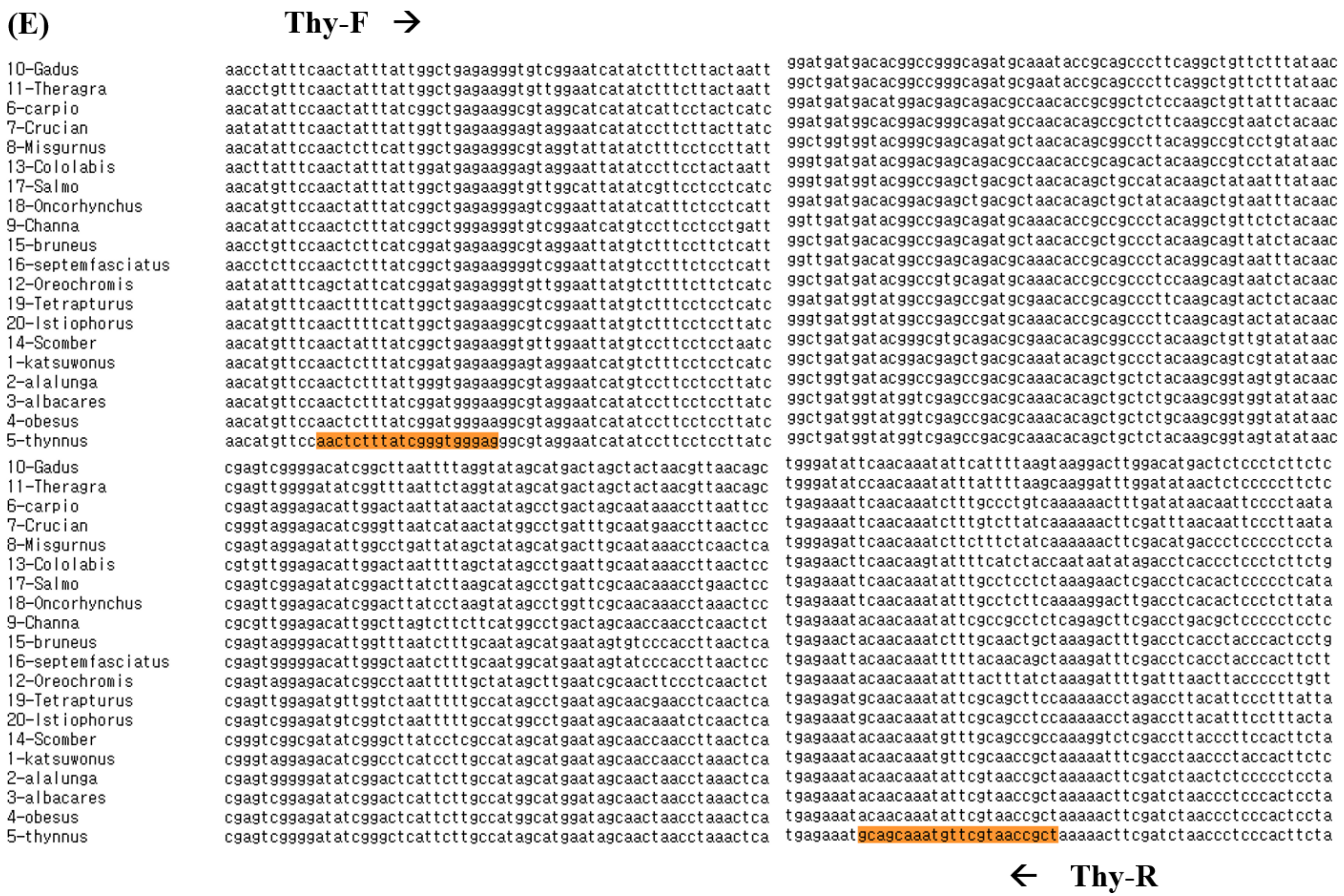


**Figure S1.** Sequence alignment of Cyb (A,B), NADH4 (C), ATP6 (D) and NADH5 (E) gene and designation of species-specific primers. Colored areas indicate the location of species-specific primers for each target. Kat (*Katsuwonus pelamis*-

specific primer), Ala (*Thunnus alalunga*-specific primer), Alba (*T.albacares*-specific primer), Obe (*T.obesus*-specific primer), Thy (*T.thynnus*-specific primer). F and R mean for Forward and Reverse primer, respectively.

**Table S1**. Specificity, accuracy and sensitivity calculated in accordance with ISO standards.

TP: the number of targets as the number of true positive values

TN: the number of non-targets as the number of true negative values.

FP: the number of false positive values

FN: the number of false negative values

(A) Specificity and accuracy of single PCR assay

| | Bigeye tuna | Skipjack tuna | Atlantic bluefin tuna | Albacore tuna | Yellowfin tuna |
|---|---|---|---|---|---|
| TP | 1 | 1 | 1 | 1 | 1 |
| TN | 19 | 19 | 19 | 19 | 19 |
| FP | 0 | 0 | 0 | 0 | 0 |
| FN | 0 | 0 | 0 | 0 | 0 |
| [1] Specificity | 100 % | 100 % | 100 % | 100 % | 100 % |
| [2] Accuracy | 100 % | 100 % | 100 % | 100 % | 100 % |

[1] Specificity: 100 x TN / (TN + FP)

[2] Accuracy: 100 × (TP + TN) / (TP + TN + FP + FN)

(B) Sensitivity of single PCR assay

| | 10 ng | 1 ng | 0.1 ng | 0.01 ng | 1 pg | 0.1 pg | 0.01 pg |
|---|---|---|---|---|---|---|---|
| Bigeye tuna | 100 % | 100 % | 100 % | 100 % | 100 % | 100 % | 0 % |
| Skipjack tuna | 100 % | 100 % | 100 % | 100 % | 100 % | 0 % | 0 % |
| Atlantic bluefin tuna | 100 % | 100 % | 100 % | 100 % | 100 % | 100 % | 100 % |
| Albacore tuna | 100 % | 100 % | 100 % | 100 % | 100 % | 100 % | 100 % |
| Yellowfin tuna | 100 % | 100 % | 100 % | 100 % | 100 % | 100 % | 100 % |

Sensitivity: 100 × TP / (TP + FN)

(C) Specificity and accuracy of multiplex PCR assay

| | Target species of multiplex PCR (Bigeye, Skipjack, Atlantic bluefin, Albacore, and Yellowfin tunas) |
|---|---|
| TP | 5 |
| TN | 15 |
| FP | 0 |
| FN | 0 |
| [1] Specificity | 100 % |
| [2] Accuracy | 100 % |

[1] Specificity: 100 x TN / (TN + FP)

[2] Accuracy: 100 × (TP + TN) / (TP + TN + FP + FN)

(D) Sensitivity of multiplex PCR assay

| | Target species of Multiplex PCR (Bigeye, Skipjack, Atlantic bluefin, Albacore, and Yellowfin tunas) |
|---|---|
| 10 ng | 100 % |
| 1 ng | 100 % |
| 0.1 ng | 100 % |
| 0.01 ng | 100 % |
| 1 pg | 100 % |
| 0.1 pg | 0 % |
| 0.01 pg | 0 % |

Sensitivity: 100 × TP / (TP + FN)
